# EEG-Based Functional Brain Networks: Does the Network Size Matter?

**DOI:** 10.1371/journal.pone.0035673

**Published:** 2012-04-25

**Authors:** Amir Joudaki, Niloufar Salehi, Mahdi Jalili, Maria G. Knyazeva

**Affiliations:** 1 Department of Computer Engineering, Sharif University of Technology, Tehran, Iran; 2 Laboratoire de Recherche en Neuroimagerie (LREN), Département des Neurosciences Cliniques (DNC), Centre Hospitalier Universitaire Vaudois (CHUV), University of Lausanne, Lausanne, Switzerland; 3 Department of Radiology, Centre Hospitalier Universitaire Vaudois, University of Lausanne, Lausanne, Switzerland; Cuban Neuroscience Center, Cuba

## Abstract

Functional connectivity in human brain can be represented as a network using electroencephalography (EEG) signals. These networks – whose nodes can vary from tens to hundreds – are characterized by neurobiologically meaningful graph theory metrics. This study investigates the degree to which various graph metrics depend upon the network size. To this end, EEGs from 32 normal subjects were recorded and functional networks of three different sizes were extracted. A state-space based method was used to calculate cross-correlation matrices between different brain regions. These correlation matrices were used to construct binary adjacency connectomes, which were assessed with regards to a number of graph metrics such as clustering coefficient, modularity, efficiency, economic efficiency, and assortativity. We showed that the estimates of these metrics significantly differ depending on the network size. Larger networks had higher efficiency, higher assortativity and lower modularity compared to those with smaller size and the same density. These findings indicate that the network size should be considered in any comparison of networks across studies.

## Introduction

Human brain is a complex system containing many interconnected regions. Among various methods for studying the brain, graph theory is a valuable framework for analyzing the anatomical and functional connectome of the brain [1], [2], [3], [4], [5]. Within the framework of graph theory, brain regions are considered to be the nodes and connection links (directed/undirected and weighted/unweighted) are extracted using some statistical measures of association.

To construct large-scale functional or anatomical brain networks, signals recorded via electroencephalography (EEG), magnetocephalography (MEG), or magnetic resonance imaging (MRI) are often used. Topological properties of such networks can be analyzed by characterizing the brain as an undirected network, where individual EEG or MEG sensors or else MRI-based regions of interests serve as nodes and a link between any two nodes represents a correlation of the time series associated with these nodes or other statistical measure of their connection [1], [6].

As network structure is extracted, it is tested for a number of neurobiologically meaningful metrics. The networks are often tested for small-worldness [7] and scale-freeness [8] – ubiquitous properties in many natural networks. Research into the brain networks has revealed their economical small-world structure characterized by high clustering (transitivity) and short average path length [9], [10], [11], [12]. Brain functional networks are cost efficient in that they implement parallel processing for low connection cost [13]. Scale-freeness has also been shown to be a property of brain networks characterized by power-law degree distribution [14], [15].

The properties of the anatomical and functional networks of brain are linked to its functions and can be affected by neurological and psychiatric diseases [16]. For example, schizophrenia patients show altered properties in functional networks obtained through EEG [17], [18] and functional MRI [19], [20]. Alzheimer's disease is characterized by abnormal small-world architecture in the structural and functional cortical networks, implying their suboptimal topological and functional organization in such patients [21], [22]. Deviant wiring of brain networks associated with the loss of visual modality and/or subsequent plastic changes was observed in early blind subjects [23].

Brain networks can be studied at both microscopic [24] and macroscopic levels [1], [2], [3], [4]. At the macroscopic scale, networks of different sizes ranging from less than 30 to 4000 nodes are extracted [18], [21], [25], [26]. However, graph metrics can be significantly influenced by the number of nodes [27]. Indeed, in a recent study, the graph metrics of structural brain networks markedly varied as a function of the network size [28].

In this paper we considered high density EEGs recorded from a number of healthy individuals and investigated how graph metrics depend on the network size. To this end, EEG-based functional networks were extracted at three different scales, and then various graph metrics were computed for the networks. We found that these metrics are significantly different across these scales at all conventional EEG frequency bands.

## Methods

### EEG recording

The EEGs of 32 healthy subjects, used for this analysis, were recorded at the Department of Clinical Neurosciences of the University of Lausanne (Lausanne, Switzerland) within the frame of projects of Dr. M.G. Knyazeva, approved by the local Ethics committee of the university (Commission cantonale d'éthique de la recherche sur l'être humain). All the procedures conformed to the Declaration of Helsinki (1964) by the World Medical Association concerning human experimentation. The participants (11 men, 21 women; mean age 51 years, standard deviation 21 years) were without substance abuse or dependence and had no known neurological or psychiatric illness or trauma. Written informed consent was obtained from all participants involved in this study.

The EEG data were collected between 11 am and 4 pm during 3–4 min of rest with eyes closed in a dedicated semi-dark room with a low level of environmental noise. To keep adequate alertness of subjects, their state and ongoing EEG were continuously monitored by experimenters. A 128-channel Geodesic Sensor Net (Electrical Geodesic Inc., Eugene, OR, USA) was used at a sampling frequency of 500 Hz. The electrode impedances were kept under 30 kΩ [29]. The sensors from the outer ring of the sensor net were not considered because of low quality signals, which left 111 sensors for analysis. The EEG time series were filtered (FIR, band-pass of 1–50 Hz and 50 Hz), re-referenced against the common average reference, and segmented into non-overlapping 1 second epochs using the NS3 software. Artifacts in all channels were edited off-line: first automatically, based on an absolute voltage threshold (100 µV) and on a transition threshold (50 µV), and then on the basis of a thorough visual inspection. The sensors producing artifacts more than 20% of the recording time were corrected using a bad channel replacement tool (NS 4.2 EGI, USA). Using short segments for analysis allowed us to record 175±69 artifact-free epochs per subject in order to achieve high confidence of the data.

To minimize the effects of volume conduction, we computed high-resolution Laplacian [30]. To this end, at each sample, a 2-D spline was fitted to common-average-reference EEG, along the surface of the best-fit sphere.

### Constructing brain functional networks

**Figure 1:**
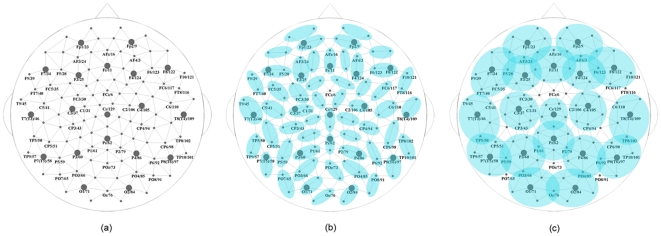
Organization of the network nodes at three different scales. The Sensor Net locations that match the positions of the International 10–10 System are labeled and followed by the numbers of the Sensor Net. The sensors corresponding to the 10–20 System are shown with grey circles. The size of the network is (a) *N* = 111, i.e., each individual sensor is used as a network node; (b) *N* = 55, i.e., pairs of sensors are used as network nodes; (c) *N* = 19, i.e., the International 10–20 System locations together with their first neighborhoods are used as network nodes (in computing S-estimator between any two group, their common nodes are removed).

For EEG-based brain functional networks, the individual (or groups of) sensors are often considered as nodes. To find connections between nodes, bivariate measures such as cross-correlation and coherence for linear dependence [31], synchronization likelihood for nonlinear association [32], and multivariate measures such as S-estimator [33] can be used. Furthermore, the EEG-based brain functional networks of various sizes can be constructed. Here we considered three networks with 111 (original number of sensors), 55 and 19 nodes (Fig. 1). Our choice was defined by the EEG montages commonly used in neuroscience research and clinical neurophysiology. The frequently applied dense-array EEG includes 111 sensors, providing a network size of 111 nodes. Another montage, traditionally used by EEG community, especially in clinical settings, is an International 10/20 system. To approximate it, we considered groups consisting of the first neighbors of 10/20 sensor locations as individual nodes resulting in a network with 19 nodes. Finally, an intermediate montage, used both in clinical and research purposes, is an Extended 10–20 system. To approximate it, we also considered a size of 55 nodes by grouping pairs of sensors.

To obtain the associations between nodes, we used S-estimator technique [33], [34], [35], which is based on entropy of the eigenvalues of the correlation matrix. For totally uncorrelated time series, dimensionality of data becomes maximized, while perfectly synchronized data lead to a minimal dimensionality [33], [34], [35]. As diversity of eigenvalues correlates with the dimensionality, it is a good measure of synchronization. Suppose we have two groups of *P*_1_- and *P*_2_-multivariate time series each with length *L*. Let us denote these time series by 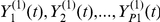 and 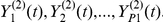 Also, let us define vectors 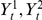 as 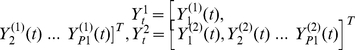. Without loss of generality, we assume that 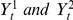 have zero mean and unitary variance. The correlation matrix is computed as
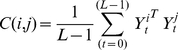
(1)and,
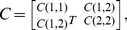
(2)Where *C(i,i)* is the internal correlation matrix of group *i*, and *C(1,2)* is the inter-correlation between groups 1 and 2. Since the correlation matrix for real time series is symmetric, we have written *C(1,2)^T^* instead of *C(2,1)*. The above correlation matrix includes both intra-group and inter-group correlations. In order to get rid of intra-group correlations, let us consider the following linear transformation [35]

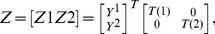
(3)Where 
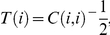
 and suppose that *R* is the correlation matrix of the transformed time series calculated as
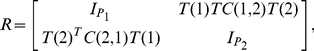
(4)where 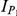 is the unity matrix of dimension *P*_1_. It is straightforward to see that after the transformation, the internal correlation of both groups is cancelled out [35]. Now, let 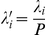 be the *i*-th normalized eigenvalue of matrix *C*, where *P* = *P*_1_+*P*_2_. Then, we compute II which is entropy of these normalized eigenvalues
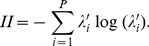
(5)


If the two groups are uncorrelated, *R*(1,2) = *R*(2,1) = 0, *R* would be diagonal, and II = log(P_1_+P_2_), whereas if they are identical, *R* will have ones on the main diagonal and zeros otherwise. The following formula has been proposed for estimating the inter-group correlation (synchronization) between these groups [35]

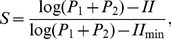
(6)where 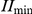 could be achieved when the time series have the lowest dimensionality, or equivalently, when they are mostly correlated. Such a quantity can be computed numerically by taking entropy of eigenvalues of matrix *A* (as obtained by Eg. (5)), which is defined as
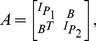
(7)where
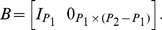
(8)


The S-estimator – as obtained through Eq. (6) – scales from 0 to 1; where 0 corresponds to completely non-synchronized systems and 1, to perfectly coherent systems.

We applied S-estimator to weight connections in cross-correlation matrices (111×111, 55×55, or 19×19). More specifically, we evaluated inter-group correlation matrices (each group containing 1, 2, or 5–8 nodes in networks with size as 111, 55, or 19 nodes, respectively, as shown in Fig. 1) by the method described above, for each epoch. For the 19-electrode case, some groups have common nodes, which were removed before computing S-estimator. By averaging these weighted matrices over all artifact-free epochs for each subject, we obtained the weighted correlation matrices, which were further used to extract the network topology. We analyzed the binary networks extracted from the weighted correlation matrices, meaning that if the weight between two nodes was larger than a threshold, the corresponding element in the adjacency matrix would be 1, otherwise, it would be 0 [1], [6], [17], [36].

**Figure 2:**
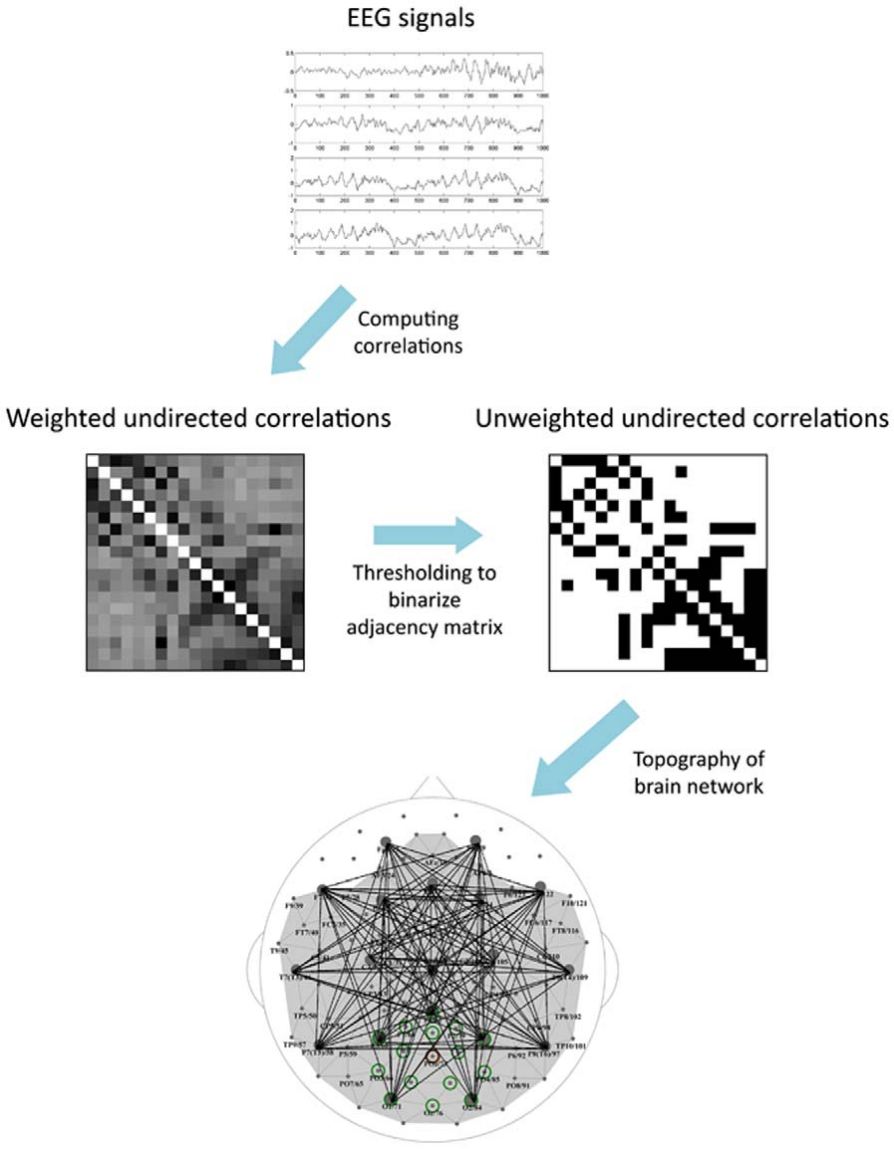
Construction of brain networks from EEG signals. The top plot shows sample EEGs taken over a time period of one second. The next step is to compute pair-wise correlations to obtain the weighted cross-correlation matrix (rows and column represent nodes). Then, the matrix is reduced to a binary form by comparing each entry with a threshold (the threshold is set such that the network has a specific density); the links with correlation values less than the threshold are set to 0; others to 1. Finally, graph theoretical metrics are calculated for the binary network.

Often, the networks are extracted for different thresholds, and then graph theory metrics are calculated for their topologies. However, this way, the networks might have different number of links and the observed differences might be due to this fact. A better way to construct the networks is based on network density [13], [19]. Here we considered the adjacency matrix *A* of an unweighted and undirected graph with *E* edges and 111, 55, or 19 nodes. We defined normalized network density (or cost) as a total number of edges in the graph *E*, divided by the maximum possible number of edges *N*(*N*−1)/2. For each subject and for a specific network cost, the weighted correlation matrices of different sizes were thresholded at different threshold values, while keeping the same density for the three extracted networks (Fig. 2).

### Graph theoretical metrics

Neurobiologically meaningful graph metrics were calculated for extracted networks. Among them, clustering coefficient and modularity refer to the processes of segregation in the brain [1], [6]. Graphs generated from real world networks usually have clusters of high density. In other words, if node A is connected to node B, and B is connected to C, A tends to connect to C. In order to quantify this phenomenon, a clustering coefficient was introduced [7]. A triplet is defined as three nodes with at least two edges, and a group is called a closed triplet, if all the nodes are connected. Clustering coefficient *C* is computed by dividing the number of closed triplets by the total number of triplets [7]

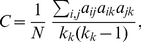
(6)where *N* is network size, *a_ij_* is the corresponding element of the adjacency matrix between nodes *i* and *j*, and *k_i_* is degree of node *i* that is obtained by summing all coming links to *i*. Clustering coefficient indeed considers local connectivity of a network by counting the neighbors of the nodes where there are links in between.

Networks' tendency to be divided into disjoint groups is also important. Nodes within a group are likely to be connected, while connections between different groups are rare. Consequently, modularity equals to the number of inter-group connections divided by the total number of edges [37]

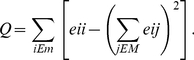
(7)Here, the network is divided into *M* disjoint modules.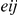 denotes the fraction of connections between modules *i* and *j*. Likewise,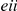 shows fraction of connections inside module *i*. The modularity index quantifies community structure of the network.

Network integration is the ability of a network to combine the information of various parts. A frequently used measure for network integration is global efficiency defined as [38]

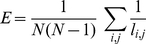
(8)where 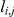 is the length of the shortest path between nodes *i* and *j*. Efficiency measures how close are the nodes in the network that is how easy the network is communicable; i.e. the higher the efficiency of a network the better communication between the nodes in the network.

Resiliency is the resistance of the network when facing random or intentional failures. It has been shown that degree-heterogeneous networks generally have high resiliency [39]. Assortativity of a network is the correlation of both sides of network edges [40]. The assortativity *r* of a network is defined as
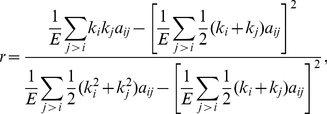
(9)where M is the number of edges. A positive value of r indicates that the network generally consists of mutually coupled high-degree nodes, while a negative assortativity implies that the network has vulnerable nodes. High-degree nodes connected to each other improve network resiliency, because they keep the nodes connected even if some links are broken.

### Statistical assessments

Wilcoxon's ranksum test was used to assess the statistically significant differences between the graph metrics of brain networks at different scales. The tests were carried out separately for all the values of network cost and the difference was considered significant at *P*<0.05.

All the computations were performed in MatLab. For Laplacian computation, we used CSD toolbox freely available at (psychophysiology.cpmc.columbia.edu/Software/CSDtoolbox). Graph theory measures were computed using brain connectivity toolbox freely available at (sites.google.com/a/brain-connectivity-toolbox.net/bct/Home).

## Results

**Figure 3:**
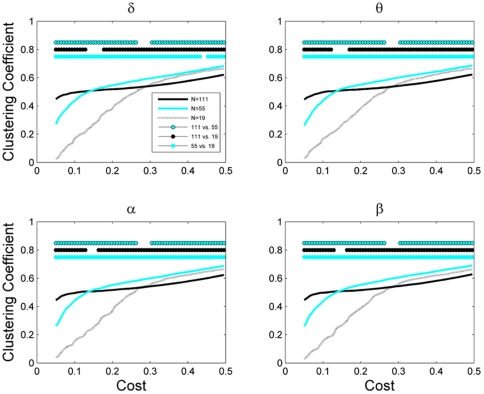
Clustering coefficient of the network as a function of network cost for different network sizes (*N* = 111, 55, and 19). Mean values of clustering coefficient are plotted for different frequency bands including delta (1–3 Hz), theta (3–7 Hz), alpha (7–13 Hz), beta (13–30 Hz). The dots above the plots represent statistically significant difference at P<0.05 (Wilcoxon's ranksum test).

**Figure 4:**
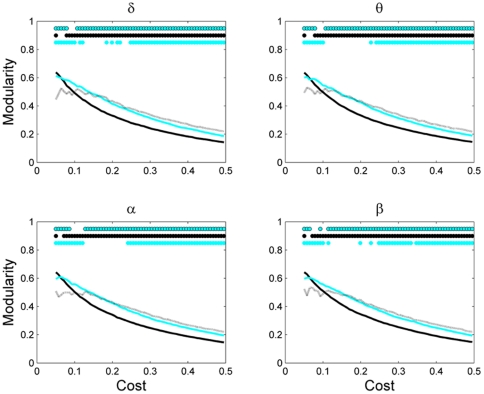
Network modularity as a function of network cost for different network sizes. Other designations are as in Fig. 3.

**Figure 5:**
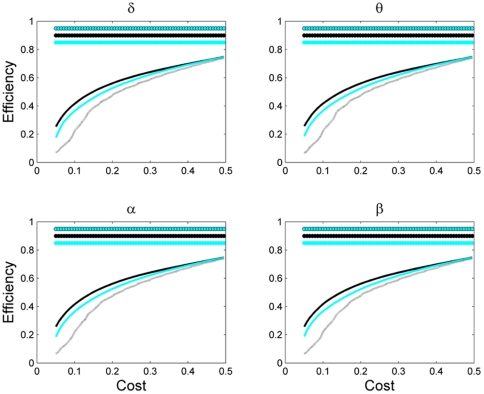
Efficiency of the network as a function of network cost for different network sizes. Other designations are as in Fig. 3.

**Figure 6:**
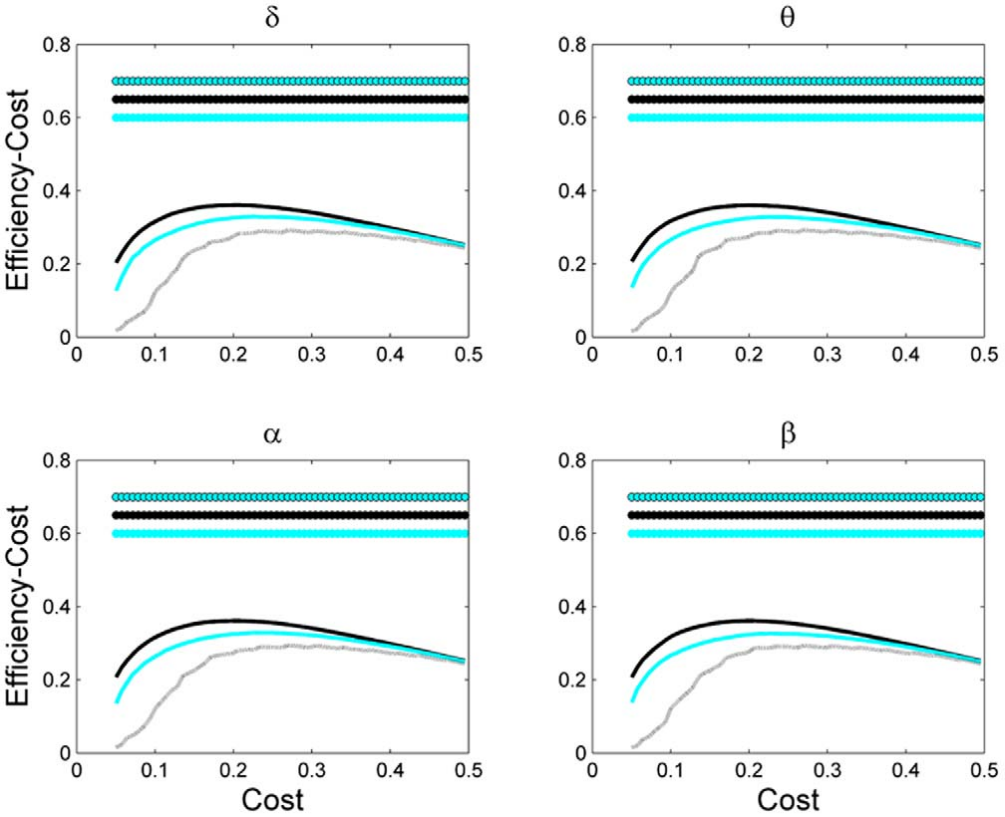
Economic efficiency of the network (i.e. efficiency minus cost) as a function of network cost for different network sizes. Other designations are similar to Fig. 3.

**Figure 7:**
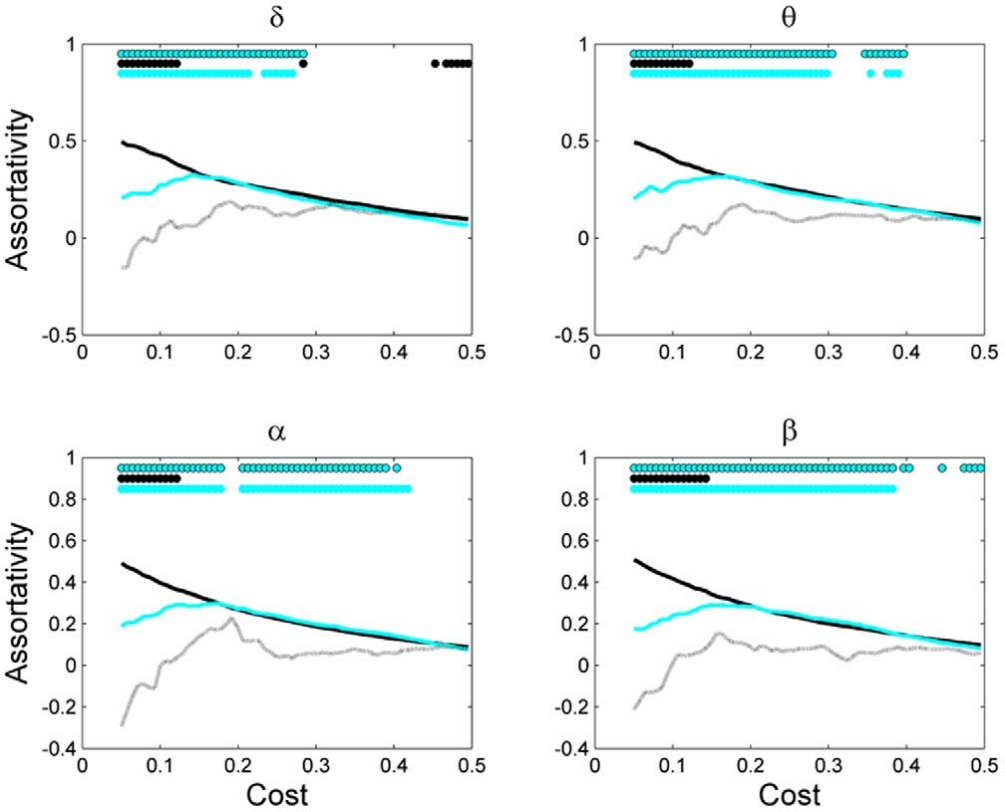
Network assortativity as a function of network cost for different network sizes. Other designations are as in Fig. 3.

We extracted the EEG functional networks at three different scales (see Methods), and calculated their properties through a number of graph metrics (Figs. 3, 4, 5, 6, 7). Fig. 3 shows the clustering coefficient as a function of network cost (density). As expected, with density increase, clustering coefficient also increased. This is due to the fact that a graph with higher density has more chance to have triangles comapred to a sparse graph. For low values of network cost, the networks with 111 nodes had significantly larger clustering than those with 55 or 19 nodes, while, for high networks costs, this was reversed (*P*<0.05, Wilcoxon's ranksum test). Networks with 55 nodes always had higher clustering than those with 19 nodes. Furthermore, the observed phenomenon was consistent across all frequency bands and the network clustering coefficient was largely dependent on its size.

Figure 4 shows the network modularity as a function of its density at different network scales. Modularity overall decreased as density increased, which is expected since dense networks consist of many noisy connections destroying the modular structure of the network, and, thus, decreasing the modularity. Similar to clustering coeefficent, for a broad range of network costs, the EEG-based brain functional networks of different sizes varied significantly in the modularity index at all frequencies.

Next, we studied global efficiency, which shows how good is the communication between nodes in the network. As the network density increased, the number of links also increased resulting in facilitating communication between its nodes, and hence, leading to the efficiency increase (Fig. 5). Efficiency of network significantly depended on its size: the smaller the netwrok, the lower the efficiency (*P*<0.05, Wilcoxon's ranksum test) for all values of cost and frequency bands.

Figure 6 shows the economic efficiency, i.e., efficiency minus cost as a function of network cost., For smaller networks, the cost of maximum economic efficiency was higher compared to a larger network. Indeed, brain network with 111 nodes had the best economic efficiency at the density values of about 0.18, while for networks with 55 and 19 nodes, these values were 0.25 and 0.32, respectively.

Finally, we analyzed the assortative behavior of these networks as a function of network cost (Fig. 7). Networks with 111 and 55 nodes always showed assortative behavior, i.e., positive assortativity coefficient. For small network costs, the assortativity of the largest network with 111 nodes was significantly higher than that of the network with 55 nodes (*P*<0.05, Wilcoxon's ranksum test). However, the network with 19 nodes had significantly different assortativity than larger networks for a broad range of network costs (*P*<0.05, Wilcoxon's ranksum test), and also showed disassortative behaviour (i.e., negative assortativity coefficient) for the small network costs.

These results indicate that the size of the EEG-based functional networks significantly influences their topological properties.

## Discussion

Graph theory tools have been recently applied to functional/anatomical brain networks constructed from time series based on MRI, MEG, or EEG. Various brain disorders have been shown to alter their properties. Examples include schizophrenia [17], [18], [19], [20], Alzheimer's disease [21], [41], and early blindness [23]. Studying the properties of brain networks in health and disease may advance us on how the brain is organized and how the disease affects this organization.

In the brain networks, nodes are considered to be brain regions and the links to represent associations between these nodes. In many real-world networks, links and nodes are well-defined. For example, in WWW, the nodes are individual web pages and the links are citations among them, or, in social networks, the nodes are individuals and the links are their acquaintances. In contrast, for the brain networks, the definition of a node depends on the recording technique. For example, in MRI-based techniques, a parcellation template is used and a region of interest is taken into account as individual node in the network [13], [15], [19]. In EEG- and MEG-based functional networks, often the individual sensor positions are taken into account as network nodes [17], [18], [36], [41]. However, different EEG techniques may have different number of sensors resulting in networks of various sizes across the studies. This raises a basic question of between-study comparability.

Using diffusion tensor imaging-based networks, it has been shown that both local and global network properties strongly depend on the parcellation scale [28]. Although the networks were small-world and scale-free, the amount of small-worldness and scale-freeness showed strong dependence on the network size [28]. For example, as size of the extracted networks increased, i.e., parcellation was performed at a finer scale, small-worldness index increased, clustering coefficient decreased, and average path length increased [28].

In this work, we analyzed properties of EEG-based brain functional networks constructed at three different scales including 111, 55, and 19 nodes. We showed significant dependence of both local and global properties of EEG-based brain functional networks upon the network size across all frequency bands. Being a local network metric, clustering coefficient showed different profiles as a function of network density at different sizes: for low network costs, the large-size network had clustering superior to the small networks, while, for high networks costs, the reverse was true. Efficiency of a network – a global network metric – is important for communicability within the network. This measure showed strong dependence on the network scale; as network size increased, while the network density was not varied, the efficiency of the network increased. The economic efficiency, defined as efficiency minus density, depended on the network scale: larger networks had optimal economic efficiency in less denser states, i.e., the larger the network the less the network cost at which the economic efficiency is optimal. Modularity and assortativity of the networks also demonstrated strong effects of scale.

In summary, while studying the properties of EEG-based brain functional networks, the network size, e.g., the number of sensors if they are considered as nodes, should also be taken into account. This work can be replicated on MEG data to investigate whether the MEG-based functional networks depend upon the network scale the same way as those reconstructed from EEG. Also, the networks that can be constructed through nonlinear inter-dependence analysis of time series are of significant interest.
